# Correlation and the time interval over which the variables are measured – A non-parametric approach

**DOI:** 10.1371/journal.pone.0206929

**Published:** 2018-11-08

**Authors:** Edna Schechtman, Amit Shelef

**Affiliations:** 1 Department of Industrial Engineering and Management, Ben-Gurion University of the Negev, Beer-Sheva, Israel; 2 Department of Industrial Management, Sapir Academic College, Sderot, Israel; Vilnius University, LITHUANIA

## Abstract

It is known that when one (or both) variable is multiplicative, the choice of differencing intervals (*n*) (for example, differencing interval of *n* = 7 means a weekly datum which is the product of seven daily data) affects the Pearson correlation coefficient (*ρ*) between variables (often asset returns) and that *ρ* converges to zero as *n* increases. This fact can cause the resulting correlation to be arbitrary, hence unreliable. We suggest using Spearman correlation (*r*) and prove that as *n* increases Spearman correlation tends to a limit which only depends on Pearson correlation based on the original data (i.e., the value for a single period). In addition, we show, via simulation, that the relative variability (CV) of the estimator of *ρ* increases with *n* and that *r* does not share this disadvantage. Therefore, we suggest using Spearman when one (or both) variable is multiplicative.

## 1. Background

In the social science literature, the connection between two variables (*X*, *Y*) is often evaluated for a specific differencing interval (*n*). It has been quite common to arbitrarily pick differencing intervals (for example, weekly (*n* = 7), monthly (*n* = 30) or quarterly (*n* = 90) data when the data available are collected daily) and use sums or products in the analyses. For example, for a sample size of 210 daily observations, a weekly differencing interval of *n* = 7 for the multiplicative case will result in 30 weekly observations: $W_{n=7,1}^{\prime },W_{n=7,2}^{\prime },\ldots ,W_{n=7,30}^{\prime }$, where $W_{n=7,j}^{\prime }=\prod_{i=(j-1)∙7+1}^{7j}X_{i}$ or, in general, $W_{n,j}^{\prime }=\prod_{i=(j-1)n+1}^{jn}X_{i}$ for *j* = 1,2,…,30. Multiplicative cases are used for rates of stocks, population size and more. However, this arbitrariness of choosing a differencing interval is dangerous as it may affect the correlation coefficient and the conclusions from the data. [1] writes: "Series can be used in correlation calculations as hourly, daily, weekly, quarterly or annual data. The resulting C/C/SV/V (Correlation, Covariance, Semi-Variance and Variance) coefficients will differ substantially for each time interval used. This is a major weakness–ideally, any accurate measure of co-movement should not be affected by the choice of time interval used for selecting variables".

It is easy to see that if two series are random walks, each consisting of the partial sums of a sequence of independent, identically distributed (i.i.d.) random variables (called additive-additive model and denoted by aa), then Pearson correlation coefficient (*ρ*_*n*_) between them will be independent of the differencing interval (*n*). However, there are cases where multiplicative models are called for. For example, growth rates of gross domestic products (GDP), industrial production, rates of stocks or population size.

For the multiplicative-multiplicative case (denoted by mm) [2] show that the limit of Pearson correlation coefficient (*ρ*_*n*_) tends to 0 as the differencing interval increases. This holds under the assumptions that both variables are positive and have positive variances (except for the case when *Y* = *kX* for some positive *k* in which the correlations are equal to 1 for all *n*) and that there is independence across time. In a recent book [3] notes that although lim_n→∞_ *ρ*_*n*_ = 0, the speed of convergence of the (Pearson) correlation matrix to the diagonal matrix is not revealed in [2]. The third case, additive-multiplicative case (denoted by am or ma, depending on which variable is taken in its multiplicative form and which one is additive), was studied by [4]. They show that Pearson correlation coefficient (*ρ*_*n*_) tends to 0 as the differencing interval increases.

[5] investigated a related problem–the effect of differencing intervals on simple regression coefficients. They illustrated that in any econometric study in which the variables have multiplicative rather than additive properties, the regression coefficients will have a mathematical bias, which is a function of the unit of time for which the empirical data are collected. [6] examined the effect of differencing intervals on multiple regression models with a combination of additive and multiplicative variables. They showed that the correlation and the partial regression coefficients approach zero as time interval increases.

The objective of this paper is twofold. The first objective is to investigate the effect of differencing interval on Spearman (*r*) correlation coefficient in the three models: additive-additive, multiplicative-multiplicative and additive-multiplicative. We adopt the assumptions used in [2] and [4], namely that the data are i.i.d. bivariate variables having second moments, and our focus is on the effect of the differencing interval. We prove that the *n*-period Spearman correlation converges to a limit as the differencing interval increases (while the *n*-period Pearson correlation converges to zero, as shown in [2] and [4]). The fact that the *n*-period Spearman correlation tends to a constant as *n* increases makes Spearman correlation a reliable measure from the theoretical point of view, because the user cannot “play” with the differencing interval to obtain a desired correlation. The second objective is to illustrate, via simulations, the median and the relative variability (CV) of the estimates for Pearson and Spearman correlations for a choice of underlying distributions.

The structure of the paper is the following: in Section 2 we present the main results of the paper, namely the limits of Spearman correlations as the differencing interval increases. In Section 3 we report on the simulation results with respect to the median and the CV of Pearson and Spearman correlations. Section 4 concludes.

## 2. The *n*-period Spearman correlation

Let (*X*_1_,*Y*_1_),(*X*_2_,*Y*_2_),… be a sequence of i.i.d. random variables having a continuous bivariate distribution that meets the conditions of the central limit theorem (CLT). Denote $E(X)=\mu_{X},\;V(X)=\sigma_{X}^{2},\;E(Y)=\mu_{Y},\;V(Y)=\sigma_{Y}^{2}$ and Pearson correlation coefficient *ρ*_*XY*_. We start with the additive-additive (aa) case. Define the random variables *W*_*n*_ = (*W*_*n*,1_,*W*_*n*,2_,…) and *V*_*n*_ = (*V*_*n*,1_,*V*_*n*,2_,…), where

$$W_{n,j}=\sum_{i=(j-1)n+1}^{jn}X_{i}$$

and

$$V_{n,j}=\sum_{i=(j-1)n+1}^{jn}Y_{i}$$

for *j* = 1,2,….

We denote their joint distribution function $F_{aa}^{\left(n\right)}(W_{n},V_{n})$. Using [7], there exists a copula $C_{aa}^{\left(n\right)}$ with uniform marginals such that

$$F_{aa}^{\left(n\right)}(W_{n},V_{n})=C_{aa}^{\left(n\right)}\left(H_{a}^{\left(n\right)}(W_{n}),G_{a}^{\left(n\right)}(V_{n})\right),$$

where $H_{a}^{\left(n\right)}$ and $G_{a}^{\left(n\right)}$ are the marginal distribution functions of *W*_*n*_ and *V*_*n*_, respectively.

Using the CLT, the distribution of the bivariate random variable

$$\left(\frac{W_{n}-n\mu_{X}}{\sqrt{n}\sigma_{X}},\frac{V_{n}-n\mu_{Y}}{\sqrt{n}\sigma_{Y}}\right)$$

tends to a bivariate normal distribution with a mean vector (0,0), a variance vector (1,1) and a correlation matrix $R=\left(\begin{array}{cc} 1 & \rho_{XY}\\ \rho_{XY} & 1 \end{array}\right)$ where *ρ*_*XY*_ is Pearson correlation coefficient between *X*_1_ and *Y*_1_. Recall that for a bivariate normal random variable with a correlation matrix *R*, the Gaussian copula can be written as

$$\Phi_{\rho_{XY}}\left(\Phi^{-1}(u),\Phi^{-1}(v)\right),$$

where Φ_*ρ*_ is the bivariate normal distribution function with correlation *ρ* and Φ is the univariate normal cdf.

Therefore, the asymptotic dependence function of

$$\left(\frac{W_{n}-n\mu_{X}}{\sqrt{n}\sigma_{X}},\frac{V_{n}-n\mu_{Y}}{\sqrt{n}\sigma_{Y}}\right)$$

is a Gaussian copula with correlation *ρ*_*XY*_. Finally, copulas are invariant under strictly increasing transformations of the marginal distributions ([8], Theorem 2.4.3) so we get the following result:

**Theorem 1:** $C_{aa}^{\left(n\right)}$ tends to Φ_*ρXY*_(Φ^−1^(*u*),Φ^−1^(*v*)).

The relationship between Pearson and Spearman correlation coefficients was studied by [9] who showed that in the case of a bivariate normal distribution,

$$\rho =2\;\sin\left(\frac{\pi }{6}r\right)$$

or equivalently

$$r=\frac{6}{\pi }arcsin\left(\frac{\rho }{2}\right)$$

where *r* is the Spearman correlation coefficient.

We now turn to the limit of the *n*-period rank correlation $r_{aa}^{\left(n\right)}$. By Theorem 1, the asymptotic copula of *W*_*n*_ and *V*_*n*_ is Gaussian. Therefore, using [9] result, the following theorem is immediate.

**Theorem 2:** $r_{aa}^{\left(n\right)}$ tends to $\frac{6}{\pi }arcsin\left(\frac{\rho_{XY}}{2}\right)$.

We note in passing that it is easy to see that for the additive-additive case, Pearson correlation is not affected by the number of periods. That is, $\rho_{aa}^{\left(n\right)}=\rho_{XY}$ for all *n*.

We now extend the result to the two other cases: multiplicative-multiplicative (mm) and additive-multiplicative (am). We start with the multiplicative-multiplicative case as in [2]. Let $(X_{1}^{\prime },Y_{1}^{\prime }),(X_{2}^{\prime },Y_{2}^{\prime }),\ldots$ be a sequence of i.i.d. random variables having a continuous bivariate distribution. Assume that $X_{i}^{\prime }$ and $Y_{i}^{\prime }$, are positive. Define the random variables $W_{n}^{\prime }=(W_{n,1}^{\prime },W_{n,2}^{\prime },\ldots )$ and $V_{n}^{\prime }=(V_{n,1}^{\prime },V_{n,2}^{\prime },\ldots )$, where

$$W_{n,j}^{\prime }=\prod_{i=(j-1)n+1}^{jn}X_{i}$$

and

$$V_{n,j}^{\prime }=\prod_{i=(j-1)n+1}^{jn}Y_{i}$$

for *j* = 1,2,….

Following the additive-additive case, the asymptotic dependence of $\left(\ln\left(W_{n}^{\prime }\right),ln(V_{n}^{\prime })\right)$ is the Gaussian copula with the dependence parameter being *ρ*_ln(*X*′)ln(*Y*′)_. The following result for the *n*-period follows [9].

**Theorem 3:** $r_{mm}^{\left(n\right)}$ tends to $\frac{6}{\pi }arcsin\left(\frac{\rho_{ln(X^{\prime })ln(Y^{\prime })}}{2}\right)$.

We note that because Spearman correlation is a rank correlation, $r(\ln\left(W_{n}^{\prime }\right),\ln\left(V_{n}^{\prime }\right))=r(W_{n}^{\prime },V_{n}^{\prime })$, which completes the proof.

Recall that [2] show that as the number of periods *n* approaches infinity, $\rho_{mm}^{\left(n\right)}$ tends to zero (except for the case when *Y* is positively proportional to *X*, in which case the correlation is 1 for all *n*).

The third case, the additive-multiplicative case follows immediately.

**Theorem 4:** $r_{am}^{\left(n\right)}$ tends to $\frac{6}{\pi }arcsin\left(\frac{\rho_{Xln(Y^{\prime })}}{2}\right)$.

The additive-multiplicative case was studied by [4]. They showed that $\rho_{am}^{\left(n\right)}$ tends to 0 as the differencing interval increases.

We note that the above theorems and proofs follow the same assumptions as in [2] and [4], namely that the data are i.i.d. bivariate variables having second moments. The focus here is on the effect of the differencing interval.

## 3. The effect of the differencing interval on the median and the relative variability (CV)

The second objective of this paper is to illustrate the effect of the differencing interval on the medians and the variabilities of the estimators of Pearson and Spearman correlation coefficients for a choice of differencing intervals via simulation. The additive-additive (aa) model will not be discussed in detail here because for this model the value of Pearson coefficient does not depend on the differencing interval. In addition, our simulation showed that in most cases its CV decreased with *n*. Therefore, Pearson correlation should be used for this model. The models under study are multiplicative-multiplicative (mm), following [2] and multiplicative-additive (ma), following [4].

In the simulation study we generated i.i.d. pairs (*X*,*Y*), choosing a normal copula where the marginal distributions for *X* and *Y* were (Normal, Normal) (both distributions are symmetric), (Lognormal, Normal) (one is symmetric and one is skewed), and (Lognormal, Lognormal) (both are skewed). The results of the three cases were similar, therefore we show the details of one case–the (Lognormal, Normal) case.

The simulation procedure is the following: In the first step we evaluated the “true” correlations. In order to do that we generated 1,000,000 pairs (*X*,*Y*), choosing a normal copula where the marginal distributions for *X* and *Y* were Lognormal(0,1.5^2^) (i.e., log(*X*) was Normal with mean 0 and variance 1.5^2^) and Normal(1,0.1^2^) (i.e., with mean 1 and variance 0.1^2^), respectively. We added 0.1 to each observation in order to move away from zero (as required by [2]). The resulting Pearson and Spearman correlations were 0.32 and 0.58, respectively.

In the second step, we generated *n* pairs of (*X*,*Y*) from the distributions described in step 1 and computed the product and sum for each choice of *n* in order to obtain a single value of the bivariate variable $\left(W_{n}^{\prime },V_{n}^{\prime }\right)$ (or $\left(W_{n}^{\prime },V_{n}^{}\right)$), according to mm (or ma) model, as detailed in section 2. The differencing intervals (*n*) were 1, 2, …, 15, 20, 25, 30, 40, 50, 75 and 100. These values were chosen in order to cover the practical ranges: weekly, monthly or quarterly intervals for daily data, where *n* = 7, 30, and 90, respectively.

In the third step, the second step was repeated *R* = 1000 times in order to obtain 1000 replications of the bivariate variable $\left(W_{n}^{\prime },V_{n}^{\prime }\right)$ (or $\left(W_{n}^{\prime },V_{n}^{}\right)$), namely $\left\{\left(W_{n,1}^{\prime },V_{n,1}^{\prime }),\ldots ,(W_{n,R}^{\prime },V_{n,R}^{\prime }\right)\right\}$ (or $\left\{\left(W_{n,1}^{\prime },V_{n,1}^{}),\ldots ,(W_{n,R}^{\prime },V_{n,R}^{}\right)\right\}$). Then, estimates of *ρ* and *r* were calculated based on *R* pairs of observations.

In the fourth step, the previous steps were repeated *M* = 2000 times to result in *M* estimates of *ρ* and *r* for each choice of *n*.

The criteria for comparison, for each *n* are the median, reported because the distributions of the estimates of Pearson correlation are skewed (as shown below), and the coefficient of variation (CV), based on *M* values for each correlation coefficient. A desirable property of a correlation coefficient is stability with respect to the differencing interval. That is, the median for *n*-period differencing should be similar to the median of the original data (i.e., the values for a single period) for all *n*, and the CV should be as small as possible.

**Fig 1:**
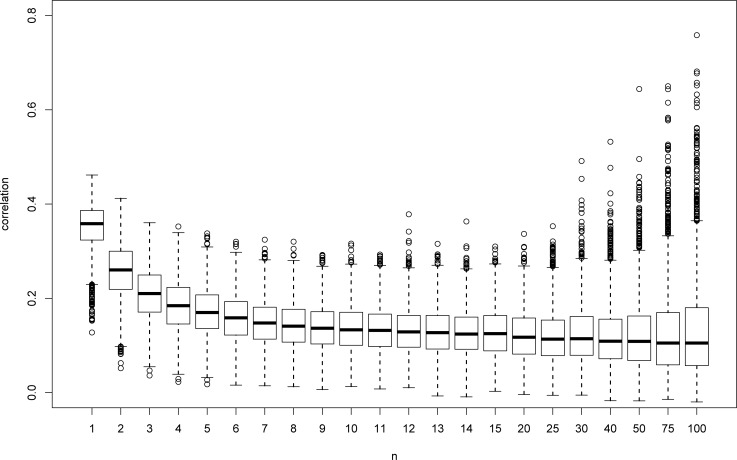
Box plots for Pearson correlations for mm model with Lognormal and Normal distributions.

**Fig 2:**
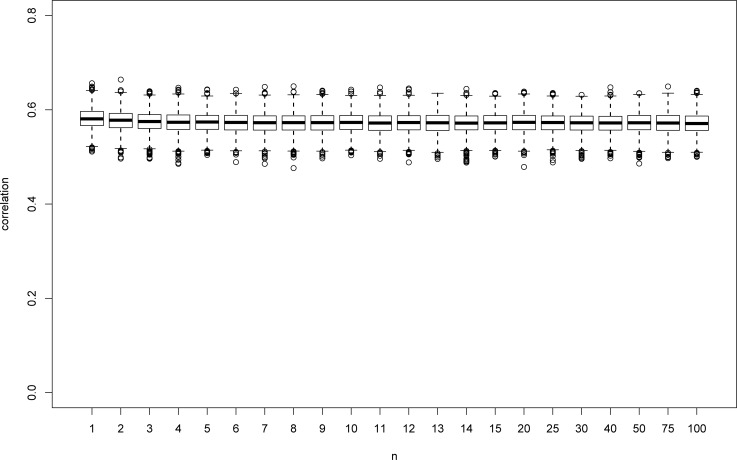
Box plots for Spearman correlations for mm model with Lognormal and Normal distributions.

**Fig 3:**
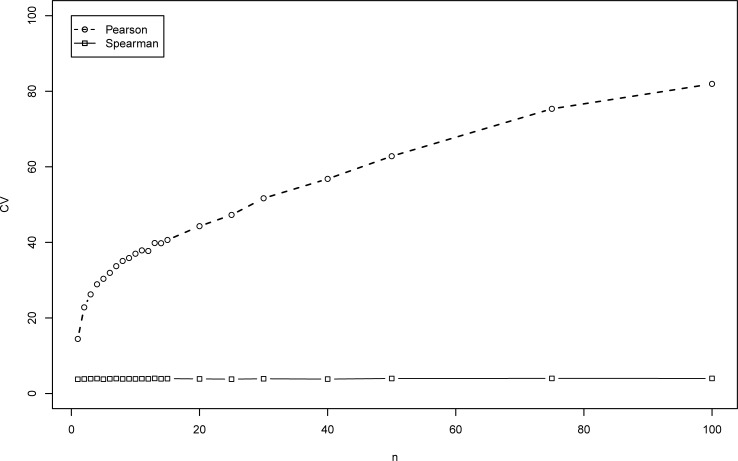
CV (in percent) of the correlation coefficients for mm model vs. *n*.

**Fig 4:**
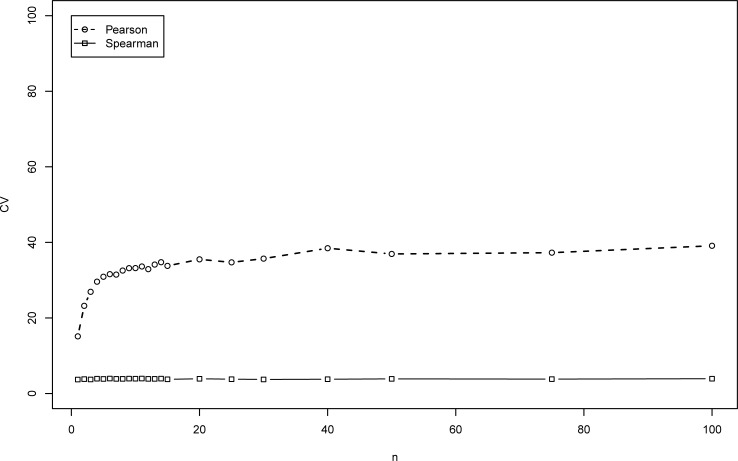
CV (in percent) of the correlation coefficients for ma model vs. *n*.

Figs 1 to 4 illustrate the comparison between the two correlation coefficients. The box plots in Figs 1 and 2 present the results for Pearson and Spearman, respectively, for the mm model. As can be seen, the median of the estimates of *ρ* decreases as *n* gets larger, as expected. Note that the median for *n* = 100 is still 0.11. However, large *n* values are needed in order to approach 0 (the value for *n* = 1500 is 0.007, not shown in the figure). As mentioned above, similar results were obtained for all cases under study. In addition, the estimates of *ρ* vary from negative values to 0.8, and the distribution becomes skewed as *n* gets larger.

The CV (in percent) is shown in Figs 3 and 4 for the mm model and the ma model, respectively. As can be seen, the CV of the estimates of *ρ* increases as *n* gets larger (up to about 80% for *n* = 100 in Fig 3, for mm model).

We now turn to Spearman *r*. As opposed to Pearson coefficient, which converges to 0, Spearman *r* converges to a known constant, given in Theorem 3. Based on 1,000,000 pairs of observations sampled from the bivariate distribution detailed above, this value is equal to 0.57 for this case. As can be seen from Fig 2, *r* converges to this value and its distribution is quite symmetric. In addition, its CV is relatively low (Fig 3) and is less than 8% for all cases under study. We note that products of random variables can result in very extreme observations. This phenomenon may have a huge impact on Pearson correlation, but only a moderate one on Spearman correlation. This fact explains the a-priori advantage of using the Spearman correlation.

The simulation study that was used for the case of homoscedasticity was repeated for the case of heteroscedasticity and the results are given in the S1 Appendix. Results in the case of heteroscedasticity were similar to the case of homoscedasticity with slight differences in the rate of convergence and in the CV, as detailed in the S1 Appendix.

## Conclusions

It is well documented that the differencing interval has an effect on Pearson correlation coefficient between two variables in multiplicative-multiplicative and multiplicative-additive models when (*X*_1_,*Y*_1_),(*X*_2_,*Y*_2_),… is a sequence of i.i.d. random variables (for the multiplicative-multiplicative model they have to be non-negative). The coefficient tends to 0 as the differencing interval increases. A natural competitor, suggested in this study, is Spearman correlation coefficient, because the distribution of the multiplicative variable is often skewed, making Pearson coefficient less suitable.

We prove that Spearman correlation converges to a limit as the differencing interval increases in the three models: additive-additive, additive-multiplicative and multiplicative-multiplicative. The limits are functions of the Pearson correlation between the original (single period) variables. In addition, we show (via simulation) that for the multiplicative-additive and multiplicative-multiplicative models the distribution of the estimate of Pearson correlation becomes skewed and its CV increases as *n* gets larger, while Spearman correlation does not share this disadvantage. In the additive-additive model Pearson correlation is constant over all differencing intervals and its CV decreases as *n* increases. Therefore, Pearson correlation is prefered for this model. The simulations were repeated for the heteroscedastic case. Results show similar patterns with slight differences.

Therefore, we suggest the use of Spearman correlation for both the multiplicative-multiplicative and the additive-multiplicative models.

## Supporting information

S1 Appendix The effect of the differencing interval on the median and the relative variability (CV) for the case of heteroscedasticity. (DOCX)
