# Supplementary material for: Correlation and the time interval over which the variables are measured – A non-parametric approach
Source: PLoS One. 2018 Nov 8;13(11):e0206929. doi: 10.1371/journal.pone.0206929 (PMC6224093; doi:10.1371/journal.pone.0206929)
Supplement: S1 Appendix — (DOCX) [file pone.0206929.s001.docx]

**S1 Appendix: The effect of the differencing interval on the median and the relative variability (CV) for the case of heteroscedasticity**

The simulation procedure is similar to the one described in section ‎3 (for the case of homoscedasticity). The main change is in the process of generating the data. Because there is no natural structure for the variances, we generated the variances using random values drawn from uniform distributions. For simplicity, we kept a constant correlation. Two cases will be presented below. In the first case the marginal distribution of $X$ is Lognormal$\left( 0,\left( unif(1,2) \right)^{2} \right)$ (i.e., log(*X*) is Normal with mean 0 and variance which is the square of a value drawn from a uniform distribution between 1 and 2). As a result, the mean of the variance used is 1.5^2^, similar to the variance used in the case of homoscedasticity. The marginal distribution of $Y$ is Normal$\left( 1 {,\left( unif(0.05,0.15) \right)}^{2} \right)$ (i.e., with mean 1 and variance which is the square of a value drawn from a uniform distribution between 0.05 and 0.15). As a result, the mean of the variance used is 0.1^2^, similar to the variance value used in the case of homoscedasticity. This procedure was repeated 1,000,000 times in order to evaluate the “true” correlations. The resulting Pearson and Spearman correlations were 0.18 and 0.57, respectively.

Figs A to D illustrate the comparison between the two correlation coefficients for the case of heteroscedasticity. The box plots in Figs A and B present the results for Pearson and Spearman, respectively, for the mm model. As can be seen, the median of the estimates of $\rho$ (Pearson, Fig A) decreases as *n* gets larger, as expected and similar to the case of homoscedasticity. In addition, the estimates of $\rho$ vary from negative values to 0.8, and the distribution becomes skewed as *n* gets larger, similar to the case of homoscedasticity. As opposed to Pearson coefficient, which converges to 0, Spearman *r* converges to a constant. As can be seen from Fig B, *r* converges to a limit when *n*=3 (in the homoscedastic case the convergence was slightly earlier, from *n*=2) and its distribution is quite symmetric, similar to the homoscedastic case. Similar results were obtained for all cases under study.


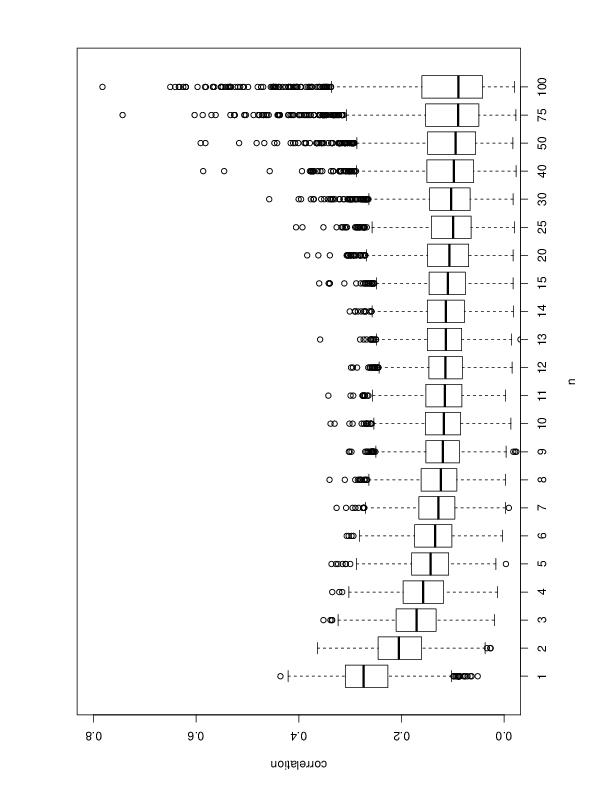


Fig A. Box plots for Pearson correlations, mm model with Lognormal and Normal distributions, case of heteroscedasticity.


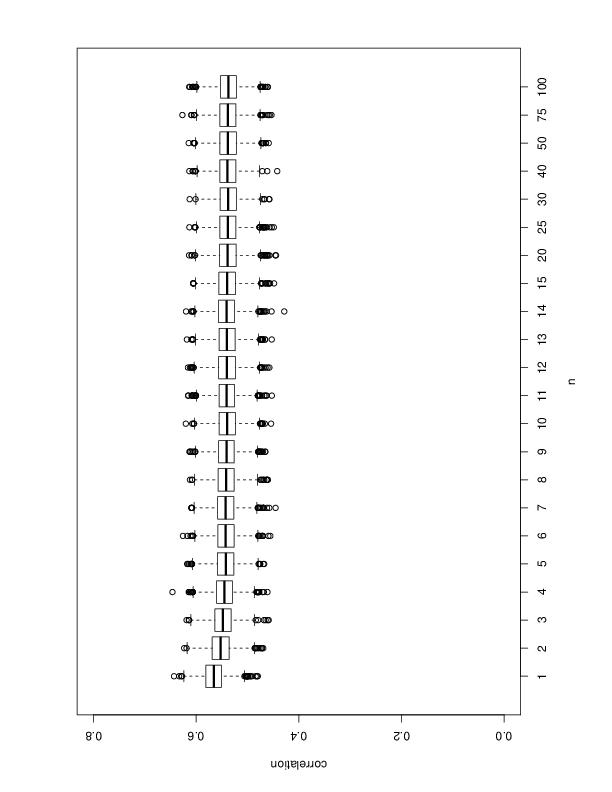


Fig B. Box plots for Spearman correlations, mm model with Lognormal and Normal distributions, case of heteroscedasticity.


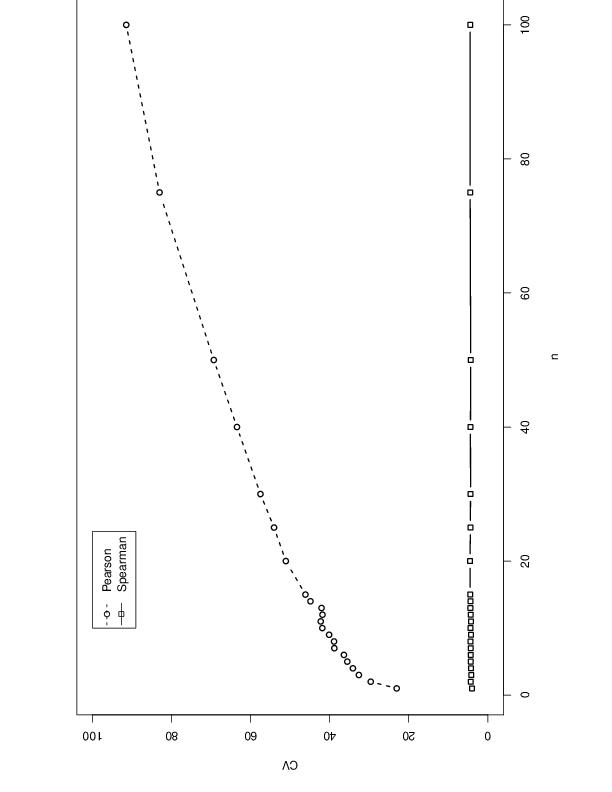


Fig C. CV (in percent) of the correlation coefficients vs. *n*, mm model, the case of heteroscedasticity.


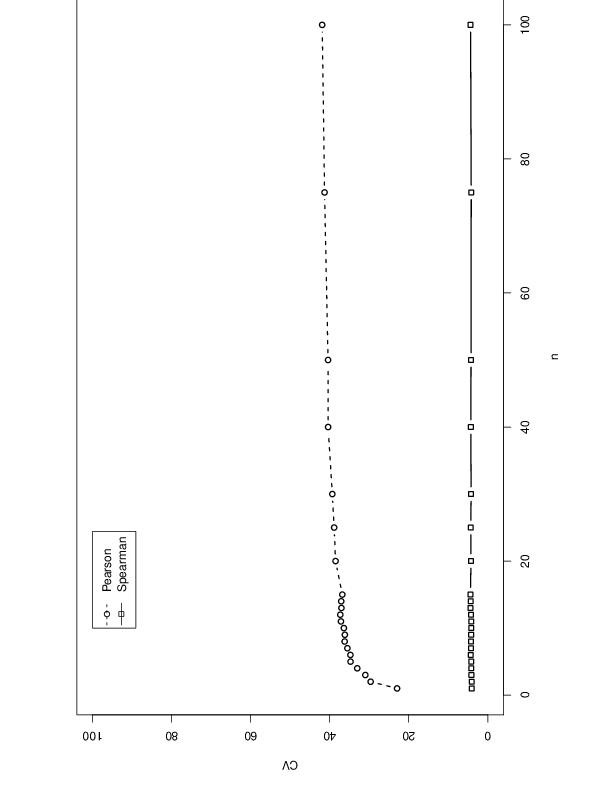


Fig D. CV (in percent) of the correlation coefficients vs. *n*, ma model, the case of heteroscedasticity.

The CV (in percent) of the estimates of Pearson and Spearman correlations are shown in Figs C and D for the mm and ma models, respectively. As can be seen, the CV of the estimates of $\rho$ for the mm model increases as *n* gets larger (up to about 90% for *n*=100 in Fig C) and is a bit higher than in the case of homoscedasticity. In the ma model the increase is moderate (Fig D), and similar to the case of homoscedasticity. The CV of *r* for the two models is relatively low (Figs C and D) and is less than 9% for all cases under study. This is similar to the case of homoscedasticity, in which the CV is at most 8%.

In order to examine the sensitivity of the performance of Spearman correlation to the magnitudes of the variances in the case of heteroscedasticity, we chose an additional (wider) range for the uniform distributions. In this case (case 2) the marginal distributions for $X$ and $Y$ were Lognormal$\left( 0,\left( unif(0,3) \right)^{2} \right)$ and Normal$\left( 1 {,\left( unif(0.01,0.19) \right)}^{2} \right)$, respectively. The means of the variances for $X$ and $Y$ are 1.5^2^ and 0.1^2^, as in the case of homoscedasticity. This procedure was repeated 1,000,000 times in order to evaluate the “true” correlation. The resulting Spearman correlation was 0.50. The box plot in Fig E presents the results for Spearman correlation for the mm model for this case. It can be seen that for this case Spearman gets closer to a limit from about *n*=5, whereas for previous cases the convergence was earlier.


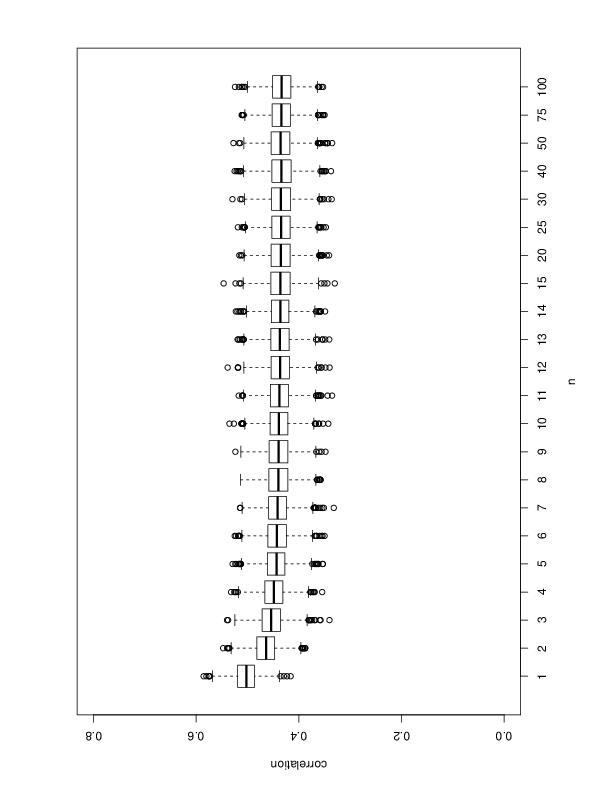


Fig E. Box plots, Spearman correlations, mm model (Lognormal and Normal), heteroscedasticity with wider range of variances.
